# Perilymph pharmacokinetics of marker applied through a cochlear implant in guinea pigs

**DOI:** 10.1371/journal.pone.0183374

**Published:** 2017-08-17

**Authors:** Alec Salt, Jared Hartsock, Ruth Gill, Daniel Smyth, Jonathon Kirk, Kristien Verhoeven

**Affiliations:** 1 Department of Otolaryngology, Washington University School of Medicine, Saint Louis Missouri, United States of America; 2 Cochlear Technology Centre Belgium, Mechelen, Belgium; 3 Cochlear Americas, Centennial, Colorado, United States of America; University of South Florida, UNITED STATES

## Abstract

Patients undergoing cochlear implantation could benefit from a simultaneous application of drugs into the ear, helping preserve residual low-frequency hearing and afferent nerve fiber populations. One way to apply drugs is to incorporate a cannula into the implant, through which drug solution is driven. For such an approach, perilymph concentrations achieved and the distribution in the ear over time have not previously been documented. We used FITC-labeled dextran as a marker, delivering it into perilymph of guinea pigs at 10 or 100 nL/min though a cannula incorporated into a cochlear implant with the outlet in the mid basal turn. After injections of varying duration (2 hours, 1 day or 7 days) perilymph was collected from the cochlear apex using a sequential sampling technique, allowing dextran levels and gradients along scala tympani to be quantified. Data were interpreted quantitatively using computer simulations of the experiments. For injections of 2 hours duration, dextran levels were critically influenced by the presence or absence of fluid leakage at the cochleostomy site. When the cochleostomy was fluid-tight, substantially higher perilymph levels were achieved at the injection site, with concentration declining along scala tympani towards the apex. Contrary to expectations, large dextran gradients along scala tympani persisted after 24 hours of sustained injection and were still present in some animals after 7 days injection. Functional changes associated with implantation and dextran delivery, and the histological state of the implant and cannula were also documented. The persistent longitudinal gradients of dextan along the ear were not readily explained by computer simulations of the experiments based on prior pharmacokinetic data. One explanation is that inner ear pharmacokinetics are altered in the period after cochlear implantation, possibly by a permeabilization of the blood-labyrinth barrier as part of the immune response to the implant.

## Introduction

Cochlear implants have been a highly successful therapy for patients with severe or profound deafness. Nevertheless, as they are an implanted “foreign body” in the ear, improvements of performance can potentially be achieved using drug or gene therapies in combination with implantation. Such therapies have potential value to preserve residual low-frequency hearing [1–7], maintain neural density [8–12], prevent endolymphatic hydrops [13], reduce fibrosis and ossification around the implant [14–15], and to maintain low electrode impedances which provide better speech discrimination [16–18]

Protocols for drug delivery in conjunction with cochlear implantation, and their influence on drug levels in the implanted cochlea, have not been studied in detail. In practice, many variables associated with drug delivery vary from study to study. The timing of drug application includes treatments before [1,2,14,19], at time of [20–21], or after cochlear implantation [22]. The route of delivery can include both systemic and local applications to the ear. Local applications include intratympanic injections, injections directly into perilymph through a cannula in the implant [23–24], or eluted into perilymph from the body of the implant [15,25].

Drug dosing has been largely empirically-based and only few studies have measured drug levels achieved in perilymph [26]. A greater understanding of perilymph pharmacokinetics for drugs delivered directly into perilymph would allow delivery protocols to be optimized to ensure efficacious, non-toxic dosing. While there have been numerous pharmacokinetic studies in animals and humans with intratympanic / round window niche applications, there are only few with intracochlear applications, either by drug injection, elution from an implant [26], or from particles placed at the time of delivery [27].

In the present study, we have measured both the drug amount and drug distribution along scala tympani (ST) following implantation with an electrode/cannula combination. The technique of “sequential perilymph sampling” was used to measure drug gradients in the ear. In this technique, 10 individual fluid samples were collected following perforation of the cochlear apex and each analyzed independently, allowing drug concentration gradients along ST to be quantified. Drug distribution and auditory function were measured at 2 hours after implantation in acute experiments, and at 24 hours or 7 days after implantation in recovery experiments. Outcome predictions and interpretation of data were performed with a computer program that simulates drug movements in the inner ear fluids.

## Materials and methods

### Animals

The study utilized 45 pigmented, NIH-strain guinea pigs weighing 400–600 g. Experiments were conducted in accordance with policies of the United States Department of Agriculture, the National Institutes of Health guidelines for the handling and use of laboratory animals, and under protocol 20140083 approved by the Institutional Animal Care and Use Committee of Washington University.

### Cochlear implantation with cannula and pump

**Fig 1:**
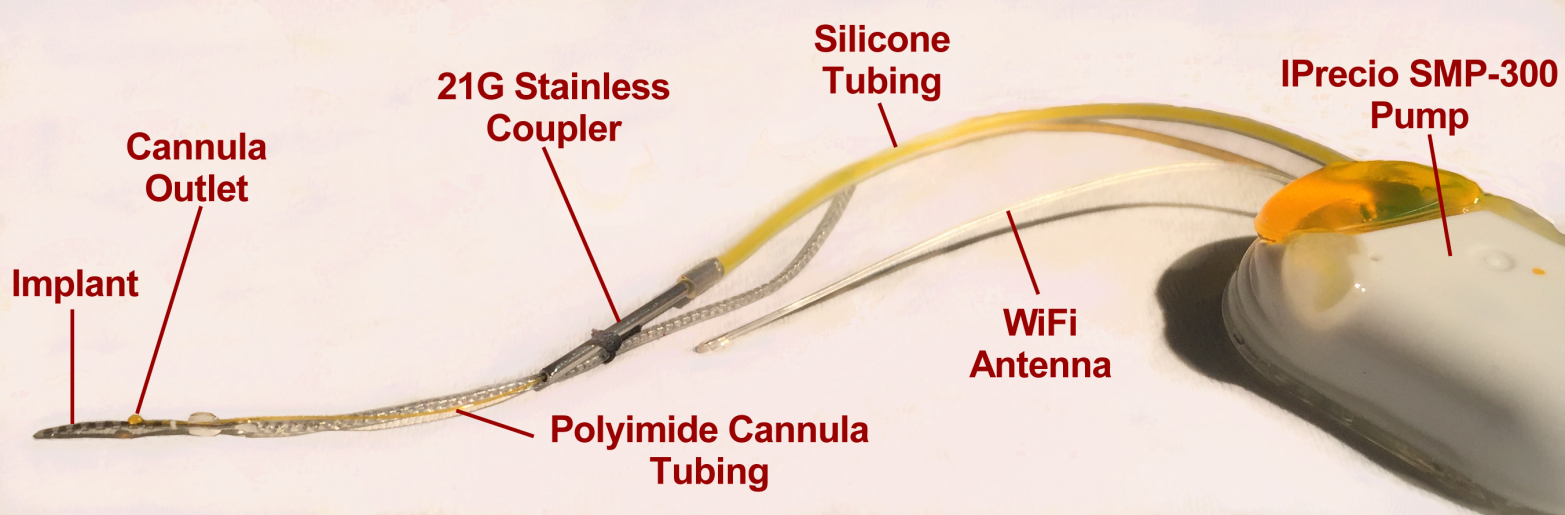
The implant-cannula-pump system ready for implantation. The pump reservoir was filled with 10 mM FITC-dextran solution in a bicarbonate-buffered artificial perilymph. The pump was coupled to the polyimide cannula of the implant with a 10 mm length of 21G stainless tubing, fixed to the polyimide tubing with cyanoacrylate. The stainless coupler was sutured to the implant to limit strain on the polyimide tubing.

The cochlear implant used in this study is shown in Fig 1. They were HL8 devices, manufactured and provided for our use by Cochlear Corp. They consisted of an 8-electrode array scaled in size to fit a guinea pig cochlea, adapted from a design by Shepherd & Xu (2002). They incorporated a 0.102 mm ID polyimide cannula, which exited the implant after the second electrode.

Cochlear implantation with continuous delivery of solution for 24 hours or 7 days was performed as a sterile, recovery procedure. Injection solution was made by adding FITC-dextran (10 mM) to sterile minimum essential medium (MEM α; 51200–038; no phenol red; Fisher Scientific: www.thermofisher.com) followed by filtration to ensure sterility. The solution was delivered from an iPRECIO SMP-300 peristaltic pump, controlled by an internal microprocessor, programmed to inject at a constant rate of 10 nl/min (0.6 μL/hr). Although the injection medium was slightly (~ 3%) hypertonic, the injection rate is so low (~0.2% of ST volume per minute) that any disturbance would be highly localized near the cannula outlet.

FITC-dextran did not stain the silicone tubing of the pump or the silicone of the implant, indicating there was no non-specific adsorption of the FITC-dextran to the delivery components of the system.

The polyimide cannula was interfaced to the 0.55 mm ID silicone tubing of the pump with a 10-mm length of 21G stainless tubing. The coupler was slipped over the polyimide and thin cyanoacrylate glue (Permabond 101) was applied to fill the space between the polyimide and the stainless tubing, without allowing it to enter the lumen of the polyimide. After the glue had hardened, the polyimide tubing extending beyond the coupler was removed with a sharp blade and the coupler was sutured to the implant to provide strain relief for the polyimide. At the time of surgery, the stainless coupler was inserted into the silicone tubing of the pump, displacing enough volume to completely fill the polyimide cannula.

For cochlear implantation as a recovery procedure, animals were initially administered 0.05mg/kg buprenorphine as an analgesic, then anesthetized with 0.8 to 1.2% isofluorane in oxygen, first in a Plexiglas chamber and then delivered through a nosecone. Heart rate and O_2_ saturation were monitored with a pulse oximeter (Surgivet, Waukesha, WI). Lidocaine (0.25–0.5 ml, 2%) was injected at the surgical site. A post-auricular incision was made, followed by opening the lateral bulla to expose the basal turn of the cochlea. A cochleostomy was drilled into ST with a slowly rotating 0.375 mm fluted carbide burr, avoiding the round window membrane. The implant and cannula system was slowly inserted into the cochlea until the silicone marker band fitted snugly into the cochleostomy, in most cases forming a fluid-tight seal. A subcutaneous pocket was made, extending from the post-auricular incision up between the scapulae. The pump was inserted into the pocket until it was located on the back of the animal. The lateral bulla was dried and closed with multiple layers of dental cement, orientating the implant and cannula tubing dorsally towards the pump. Muscles and skin were sutured closed. Animals were given subcutaneous lactated Ringer’s solution and 0.05 mg/kg buprenorphine every 8–12 hours.

### Animal preparation for short injections and perilymph sampling

All animals underwent a terminal procedure in which inner ear fluids were sampled for analysis and in some, auditory function was also tested. Animals were anesthetized with 100 mg/kg sodium thiobutabarbital (Inactin, Sigma, St Louis, MO) and maintained on 0.8 to 1.2% isofluorane in oxygen using a mechanical ventilator combined with a tracheal cannula. A 5% end-tidal CO_2_ level was maintained, monitored with a CapnoTrue AMP (Bluepoint Medical, The Netherlands), through adjustment of the ventilator’s tidal volume. Heart rate and oxygen saturation were monitored with a (Surgivet. Waukesha, WI) pulse-oximeter. Body temperature was maintained at 38°C with a thermistor-controlled heating blanket. At the end of the procedure, animals were sacrificed while under deep anesthesia by either intravenous injection of 3M KCl or by exsanguination during the removal of temporal bones.

In those animals that had not been injected by the recovery procedure described above, a single, 2 hr injection was performed. In these experiments, injection rates of either 10 nL/min or 100 nL/min were used, performed with an iPRECIO SMP-300 pump (10 nL/min; 130 μL reservoir, available rates 0–167 nl/min), an iPRECIO SMP-200 pump (100 nL/min; 900 μL reservoir, available rates 16–500 nl/min) or with a World Precision Instruments Ultrapump (motor driven syringe pump) at 10nL/min or 100 nL/min. The injected solution was 1 or 10 mM FITC-dextran (fluorescent dextran, FW ~4000, Sigma-Aldrich, St. Louis MO) in a background artificial perilymph solution containing NaCl (125 mM), KCl (3.5 mM), NaHCO_3_ (25 mM), CaCl_2_ (1.3 mM), MgCl_2_ (1.2 mM), NaH_2_PO_4_ (0.75 mM), and dextrose (5 mM).

### Sequential sampling from the cochlear apex

**Fig 2:**
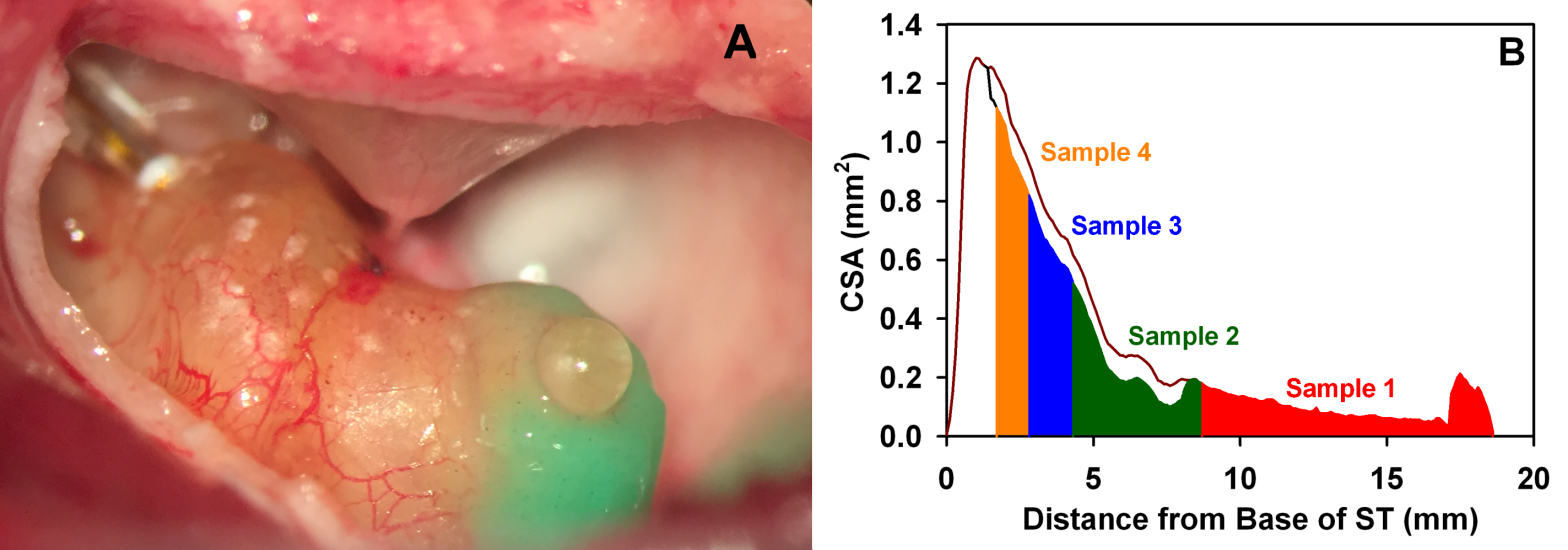
Perilymph sample origins. (A) Exposed guinea pig cochlea with a cochlear implant inserted into the basal turn of scala tympani and with the apex prepared for sequential fluid sampling. The green substance surrounding the apex is silicone adhesive (see methods). The apex has been perforated and fluid efflux is accumulating on the hydrophobic surface, which can be collected with minimal contamination. (B) Calculated spatial origins for the first four 1 μL perilymph samples collected from the apex, based on the measured cross-sectional area (CSA) of the perilymphatic compartments. The first sample includes the apical portion of scala vestibuli where the samples are collected. Later samples take into account the unavailable volume occupied by the cochlear implant in the 1.3 to 8.3 mm region of scala tympani (ST). Fluid samples are also influenced by solute exchange with adjacent compartments as they travel up the cochlea (not represented in this figure).

Gradients of dextran along the perilymphatic spaces were measured directly from multiple samples obtained by a technique called “sequential sampling” ([28,29]. When the apex is perforated, perilymph is driven out by cerebrospinal fluid (CSF) entering the basal turn of ST through the cochlear aqueduct, pushing perilymph in an apical direction along the scala. The first sample collected originates from perilymph near the apex and each following sample from perilymph that originated from a scala location progressively closer to the base. After all ST perilymph has been pushed out, subsequent samples contain CSF that has passed through the scala. Samples collected in this manner allow drug gradients along the length of ST to be quantified. Perilymph was collected from the cochlear apex as a series of individual 1 μL samples collected over a 10–20 min period. To prepare the cochlea for sample collection the middle ear mucosa overlying the cochlear apex was first removed and the bone was allowed to dry. A thin layer of cyanoacrylate glue (Permabond 101; Permabond, Pottstown, PA) was applied to the dry bone, followed by layers of two-part silicone adhesive (Kwik-Cast, World Precision Instruments, Sarasota, FL), built up at the edges to form a hydrophobic cup. At the time of sampling a 30–40 μm fenestration was made at the apex through the adhesives using a 30° House stapes pick (N1705 80, Bausch and Lomb Inc.). Clear, uncontaminated fluid flows from the fenestration, accumulating on the hydrophobic surface, as shown in Fig 2A. Fluid was collected with hand-held, blunt tipped capillary tubes (VWR 53432–706; VWR Radnor, PA), each marked for a nominal volume of 1 μL and taking 1–2 min to collect. The length of each sample in its capillary tube was measured with a calibrated dissecting microscope, from which the exact sample volume was established. Ten individual samples were collected in this manner. The approximate spatial origins of the first four fluid samples, based on the cross-sectional area of the guinea pig ST and corrected for the area occupied by the cochlear implant, are shown in Fig 2B. Data derived from direct measurement of sample concentrations are shown as solid symbols in the figures.

### Sample handling and analysis

Samples from the ear and samples of the injection medium were expelled from the collection capillary into 150 μL of diluent (phosphate-buffered saline with sodium azide preservative (Santa Cruz Biotechnology, Inc; www.scbt.com). Injection solution was also used to make a dilution series, by adding 300 μL of solution to 2.7 ml PBS diluent (10x dilution), repeated 8 times. Samples were loaded into a disposable 96 well plate and read in a SpectraMax i3 plate reader with SoftMax Pro 6.4 software with parameters optimized for FITC-dextran (495 nm excitation, 15 nm bandwidth and 535 nm emission, 25 nm bandwidth). A sigmoid curve (Hill function) was fitted to the dilution series measurements and was used to convert fluorescence brightness to concentration.

### Data interpretation through simulations of the experiments

Predictions and quantitative interpretation of measurements were provided by our established simulation program, available for download at oto.wustl.edu/saltlab. The simulator calculates solute distribution based only on established physical processes, such as diffusion, volume flow, elimination and exchange with adjacent compartments. Simulations take into account the dimensions all the fluid and tissue spaces of the guinea pig cochlea and vestibular system. The cross-sectional area of each compartment is defined with 0.1 mm resolution along its length, derived from 3D reconstructions of orthogonal plane fluorescence optical sectioning (OPFOS) images for 16 compartments of the ear. In this application, the simulations corrected the ST area with distance based on the space occupied by the implant. ST kinetics are also corrected for the volume change caused by the implant, as a specific transport process acting into a smaller fluid volume results in faster kinetics. The kinetic parameters used for FITC-dextran distribution in the cochlea were based on those derived from analysis of 10 different FITC-dextran application and sampling conditions [30]. Data derived from simulations are shown as open symbols in the Figures.

### Functional measurements

Sound stimulation and response collection were performed with Tucker-Davis system 3 hardware, controlled by a custom-written data collection program. Sounds were delivered in a closed system through a hollow ear bar, placed in the external canal after it had been transected.

Cochlear action potential (CAP) responses were measured from a ball electrode placed on the cochlear apex. The apex was chosen to avoid local influences of the implant, such as additional fluid accumulation at the RW niche due to fluid leakage at the cochleostomy. CAP responses were collected in response to 20 alternating tone burst stimuli. For frequencies > 4 kHz we used a 0.5 ms linear rise-time and for frequencies < 4 kHz a linear, 2 cycle rise time (i.e. 4 ms at 500 Hz). Responses were low-pass filtered with a 2-kHz cutoff for stimuli > 2 kHz and with the cutoff at the stimulus frequency for responses to stimuli below 2 kHz. The filtering was intended to attenuate cochlear microphonics and more importantly auditory nerve overlapped waveform (ANOW) response components [31] that occur at twice the stimulus frequency. For responses to stimuli of 3 kHz and above, CAP was measured as the amplitude of the N_1_-P_1_ component. For CAP responses to stimuli of less than 3 kHz, AP transitioned into a slow negative wave which was measured as the baseline-N_1_ amplitude. CAP thresholds were established using an automated procedure with 10 μV criterion, increasing sound level in 5 dB steps until an above-criterion response was detected, then decreasing in 5 dB steps. With these procedures to minimize CM and ANOW contributions at low frequency we were able to record AP thresholds down to 200 Hz. Typically, cochlear sensitivity was measured from 200 Hz to 22 kHz in ¼ -octave steps.

Acoustic emission (2f_1_-f_2_) thresholds were measured using an Etymotic ER10C system incorporated into the hollow ear bar. F_2_ was set to a frequency 1.2x f_1_ and to a level 10 dB below f_1_. Stimulus levels were adjusted in 5 dB steps until the 2f_1_-f_2_ emission was 10 dB above the unstimulated noise floor. Emission thresholds were collected from 500 Hz to 8 kHz in ¼-octave steps.

### Histological assessment

The ear of animals undergoing 24 hr and 7 day injections were fixed for histological assessment. The implant was left in place and was cut off with fine scissors just outside the cochlea at the cochleostomy. The ear was isolated, the round window perforated and the stapes dislocated and was fixed by immersion in 4% paraformaldehyde + 0.1% glutaraldehyde in phosphate buffered saline. Ears were subsequently either microdissected under water or dehydrated in ethanol and embedded in Epotek epoxy resin (www.epotek.com). Resin embedded specimens were surface-ground and surface-stained with eosin Y and toluidine blue to reveal internal structures in relation to the implant.

Statistical significance was assessed by Sigmaplot v13 software (Systat: systatsoftware.com)

## Results

### Two hour injection studies

**Fig 3:**
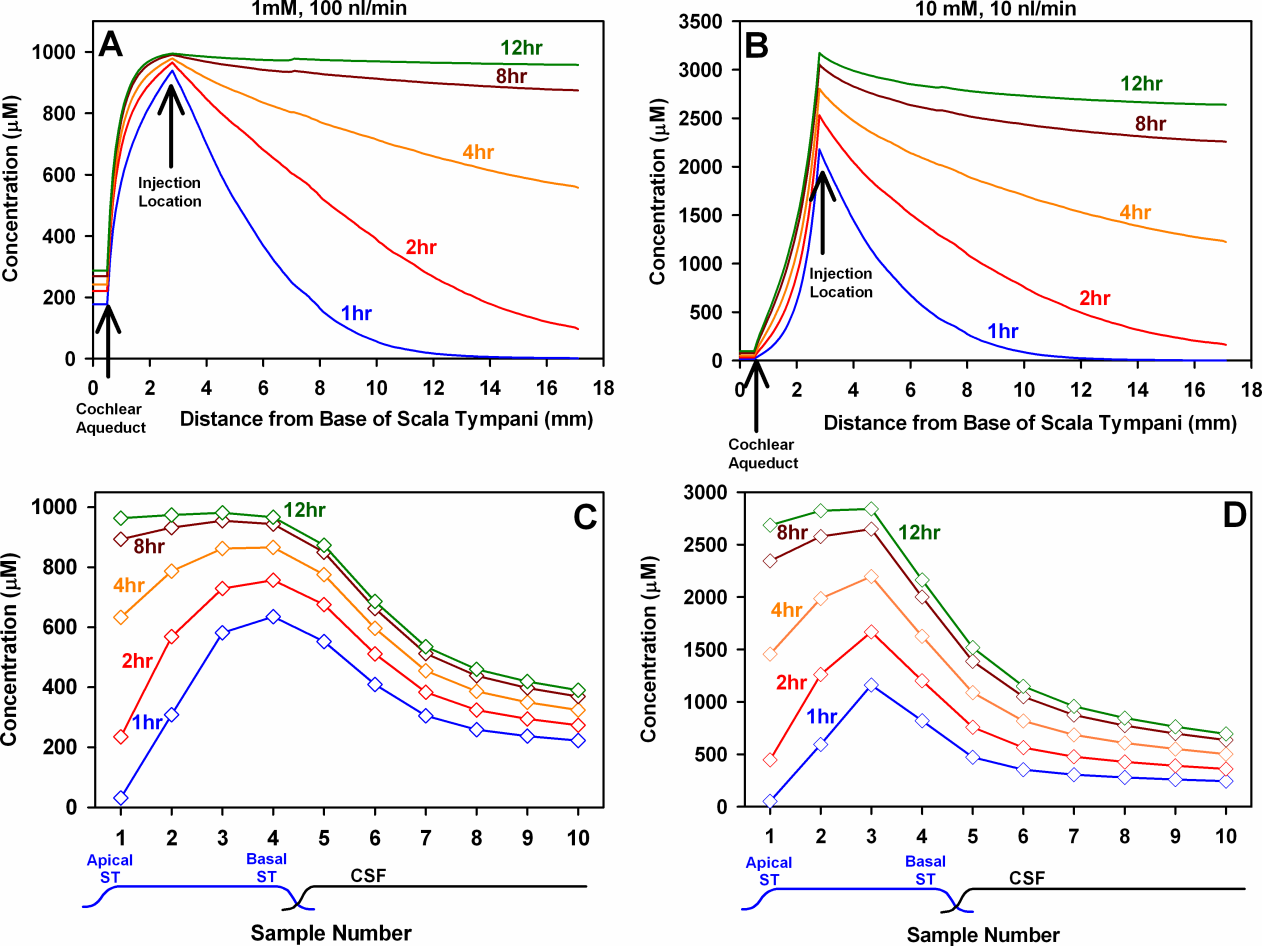
Calculated gradients and sample concentrations. Calculated gradients along ST (upper row) and corresponding predicted sample concentrations (lower row) for FITC-dextran injections into ST for durations of 1 to 12 hours. The left column shows the results of 1 mM dextran injected at 100 nL/ min, and the right column shows the results of 10 mM dextran injected at 10 nl/min, with both injections occurring at 2.8 mm from the base of ST. The calculations use simulator parameters based on analysis of FITC-dextran data for 10 different injection-sampling conditions [30], summarized in the text. Initially, a large gradient for dextran along ST is expected (red and blue curves in A and B) but the gradient is expected to decline as injection is prolonged and to disappear after 8–12 hours injection. The same is expected with the lower injection rate, but in this case the basal region of ST is not filled as effectively with dextran (B vs. A), so the peak of the sample curve occurs at sample 3 with 10 nL/min injection (D), rather than sample 4 with 100 nL/min injection (C).

The predicted distribution of FITC-dextran following local injections into the basal turn of ST was calculated, as shown in Fig 3. The simulation parameters were based on extensive prior studies in which FITC-dextran marker was injected into perilymph and sampled with 10 different protocols. Simulation parameters were established that fitted the measured data from all 10 protocols [30]. In those experiments, dextran was injected from glass pipettes sealed into the bony wall of the otic capsule, with perilymph sampling occurring up to two hours afterwards. Fig 3 shows the calculated concentrations two injection rates (100 nL/min and 10 nL/min). The distribution of dextran along the length of ST (Fig 3; upper row) and the calculated sample concentrations for 10 x 1 μL samples collected sequentially from the cochlear apex (Fig 3; lower row) are shown for high rate, low concentration (100 nL/min, 1 mM) and for low rate, high concentration (10 nL/min, 10 mM) injections from the cochlear implant of varying durations from 1 to 12 hours. The concentration distribution along ST is initially dominated by the basal turn application, with concentration declining markedly with distance towards the apex. With time, the apical concentration is predicted to rise as the dextran spreads both by diffusion and under the influence of a small apically directed flow (~30 nL/min) shown to exist in the normal, sealed cochlea. The corresponding samples that would result from the drug distributions in Fig 3A & 3B are given in Fig 3C & 3D respectively. For both injection conditions the first sample, originating from apical perilymph, is initially low but increases over time as dextran spreads apically with time along the cochlea. Although samples 4–5 originate from perilymph at the base of ST, they pass along the length of ST, interacting with adjacent tissue compartments, so the concentrations of samples 4 and 5 are lower when there is lower concentration in apical regions. The simulations incorporate and take into account the inter-compartment interactions during delivery and sampling. The simulations predict that substantial gradients of dextran along the cochlea will occur at early times (such as 1 or 2 hours) after injection starts. The calculated ratio of sample 3 to sample 1 (hereafter described as the 3:1 ratio) after 2 hrs injection was 3.10 at 100 nL/min injection rate and 3.62 at 10 nL/min injection rate. Previous studies have shown that dextran is retained well in perilymph (i.e. elimination rate is low), so the dextran would be expected to distribute to the apical regions within 8–12 hrs of injection, with a 3:1 ratio then approaching 1.

**Fig 4:**
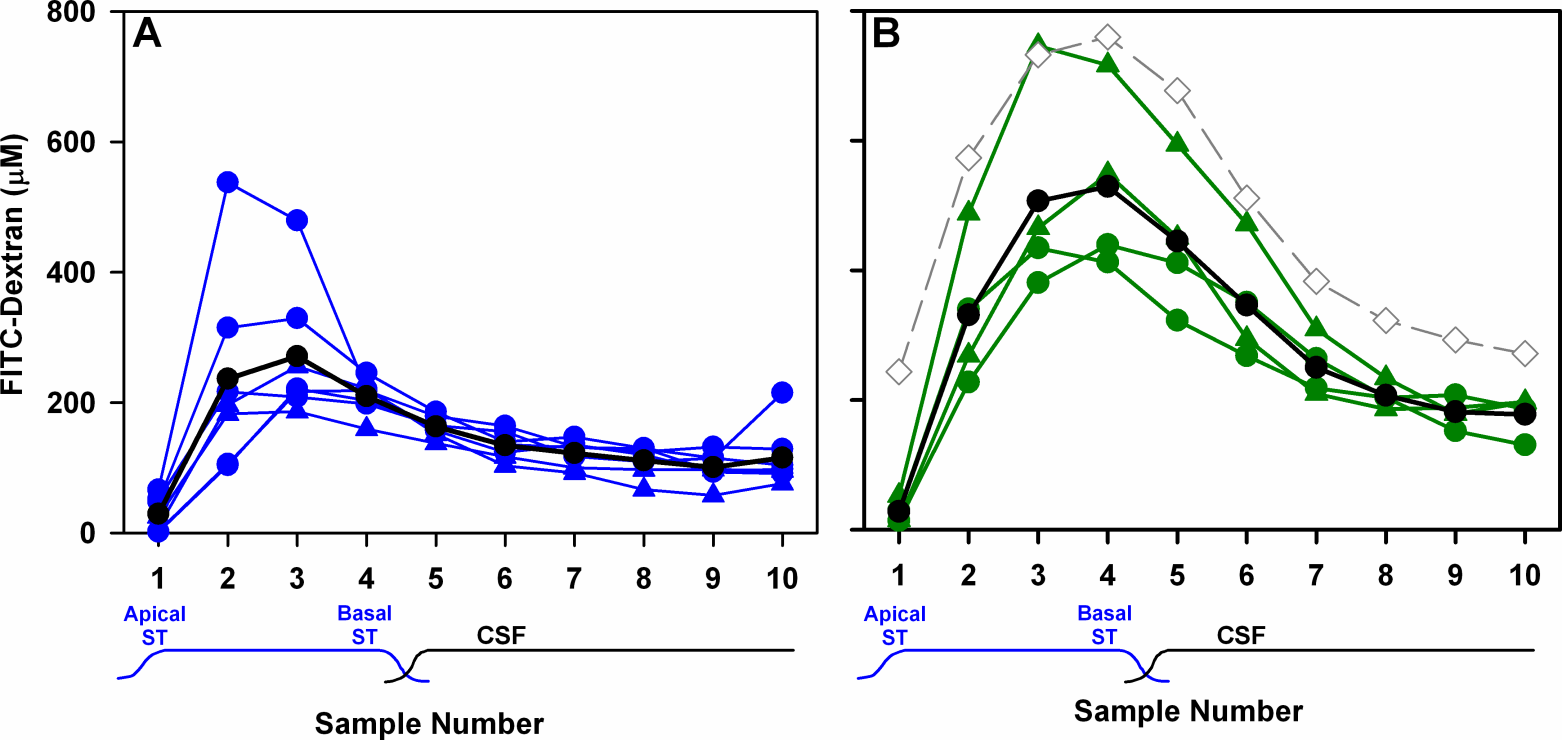
Measured dextran concentrations in perilymph samples. Dextran concentrations were measured for sequential fluid samples collected from the apex of implanted animals in which the cochleostomy was leaking during injection (A) or in which there was no detectable leak from the cochleostomy during the 2 hr injection period (B). Injections were performed at 100 nL/min either with a WPI Ultrapump (solid circles) or with iPRECIO SMP-200 pumps (solid triangles). Group average curves are shown in black. With leakage at the cochleostomy, the peak of the average marker concentration occurred in the 3^rd^ sample and averaged 271 μM (SD 103, n = 7) while with no leakage at the cochleostomy the peak of the average marker concentration occurred in the 4^th^ sample and averaged 529 μM (SD 138, n = 4). The predicted curve (from Fig 3C; 2 hr) is shown for comparison (gray diamonds). Measured concentrations were typically lower than predicted.

The results of perilymph sampling experiments following 2 hr injections at 100 nL/min are shown in Fig 4. Based on years of experience trying to seal measurement and injection pipettes into perilymph, we initially expected it to be impossible to seal something the size of the cochlear implant effectively into the otic capsule. As low rates of leakage would be difficult to measure, we classified the amount of leakage based on the state of the middle ear at the end of the two-hour injection. If there was fluid leakage at the cochleostomy, fluid typically leaked at about 1 μL/min, which amounted to 120 μL over the 2 hr period. Such a volume would almost fill the bulla so it would be easily recognized. Based on this criterion we found that packing around the implant with fascia and/or bone dust was not able to produce a fluid-tight seal. We subsequently discovered that when the cochleostomy was carefully drilled with a slowly-rotating burr, without entering the scala, it could be precisely sized so that the implant would fit tightly. Furthermore, as the implant incorporated a raised silicone band as an insertion marker, we found it was possible to insert the compressible insertion band snugly into the cochleostomy, to an extent that a complete fluid seal was achieved. Experiments were subsequently classified more rigorously based on whether **any** fluid accumulation occurred in the RW niche during the 2 hr experiment. Those in which we found no fluid in the niche were classified as having a sealed cochleostomy. If any fluid was present after 2 hrs, the experiment was classified as having a leaking cochleostomy. In practice, intermediate, low rates of leakage were not found to occur. Either the cochleostomy leaked profusely, or it was sealed. The concentration of FITC-dextran in perilymph after 2 hrs injection was highly dependent on whether the cochleostomy leaked or not, as shown in Fig 4. When there was leakage at the cochleostomy the peak of the group mean sample concentration curve (Fig 4A; black curve) occurred in sample 3 and averaged 271 μM (SD 103, n = 7). In comparison, with no leakage at the cochleostomy the peak of the average marker concentration was significantly higher (2-way ANOVA, Bonferroni p<0.001). The peak of the group mean sample curve occurred in sample 4 (Fig 4B; black curve) and averaged 529 μM (SD 138, n = 4).

Even in the absence of leakage at the cochleostomy, most of the measured perilymph concentration curves were below predicted concentrations (shown in Fig 4B as the gray dotted line, taken from Fig 3C, 2 hr curve). Thus, in the injected, implanted ear we generally found lower perilymph concentrations of FITC-dextran compared to injections from glass pipettes sealed into the perilymphatic space as performed in our prior study and on which the predictions were based. In each of the concentration curves in Fig 4B, the measured concentration of sample 1 was notably lower than that predicted by simulation, with calculated 3:1 ratios of the measurements averaging 22.0 (SD 8.8, n = 4).

### 24 hour and 7 day injection studies

**Fig 5:**
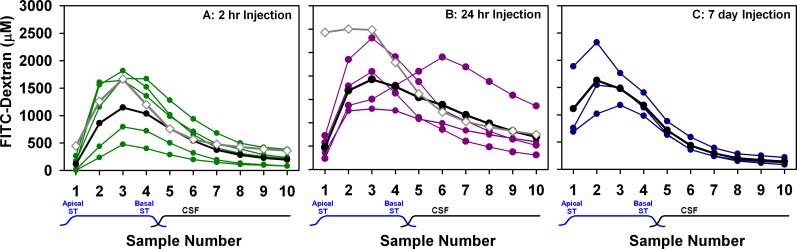
Dextran measurements at three time-points. Perilymph concentration of FITC-dextran in sequential samples collected from the apex after 2 hr (A), 24 hr (B) or 7 day (C) injections at 10 nL/min. Colored curves are individual experiments, black curves are the group mean and gray curves with open symbols are the simulator predictions. The mean 2 hr curve is lower, but similar to the predicted curve, with sample 3 having the highest concentration. Contrary to the prediction, dextran gradients were found to be still present even after 24 hours of injection, as seen in the low concentration found in sample 1 of all animals relative to sample 3. Even after 7 days, gradients still remained in some animals.

For prolonged FITC-dextran injections through the implant we chose to use the iPRECIO SMP-300 pump which has a 130 μL reservoir. At an injection rate of 10 nL/min, this pump will run for 9 days without re-fill. The results of sampling perilymph in animals after 2 hr, 24 hr and 7 day injections of FITC-dextran at 10 nL/min are summarized in Fig 5. For 2 hr injections (Fig 5A) the peak concentration occurred in sample 3, consistent with the simulator prediction (Gray curve shows prediction from Fig 3D, 2 hr). In 3 animals, the peak was close to the predicted concentration, but was lower in the other 2, so the mean curve (black) was lower than the prediction. In similarity with the 100 nL/min injections, measured sample 1 concentration was lower than the simulator prediction. The 3:1 ratio for the measured 2 hr curves averaged 57.4, but was highly variable (SD 64.7, n = 5).

After 24 hr injections (Fig 5B), perilymph concentration was significantly higher (2-way ANOVA, Bonferroni t-test, p<0.001), in keeping with the prediction. Nevertheless, substantial gradients remained, as demonstrated by the 3:1 ratio for the measured 24 hr curves which still averaged 4.40 (SD 1.4, n = 4). In one animal, an abnormally late peak was observed at sample 6.

**Fig 6:**
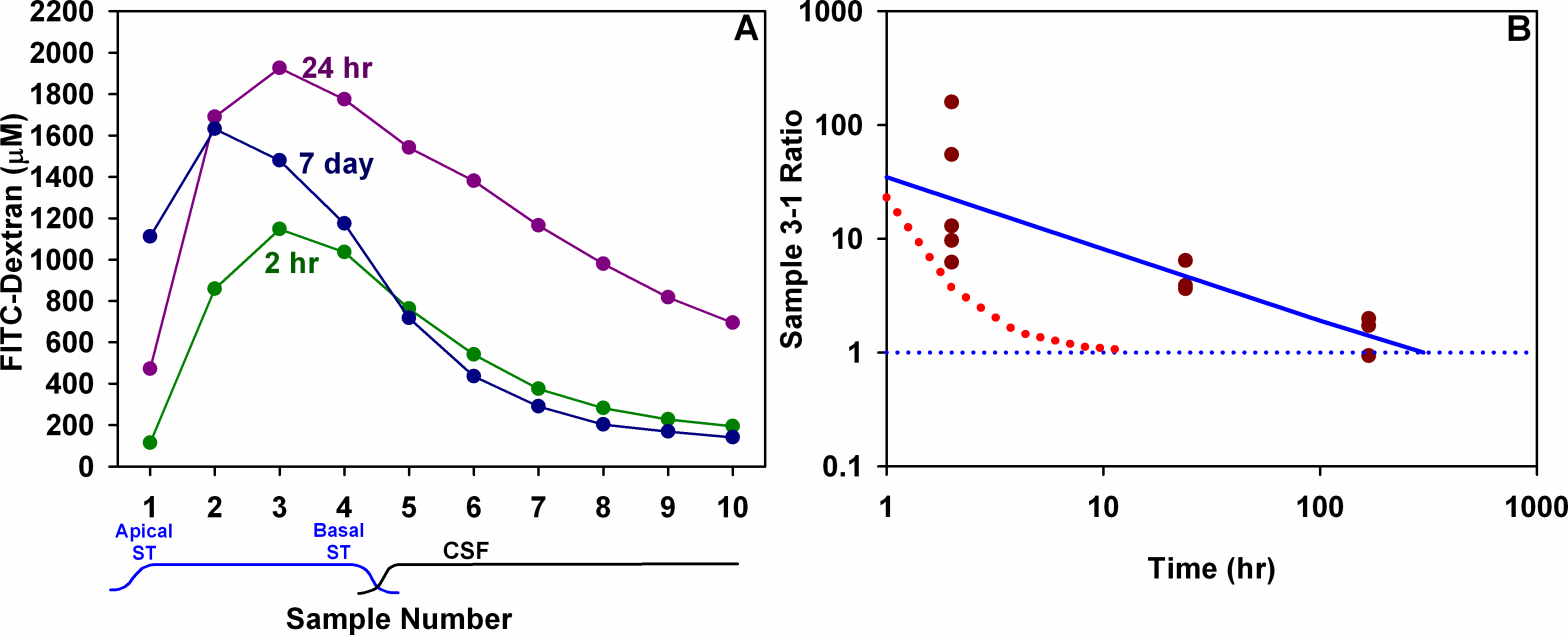
Perilymph measurements at 3 time points compared. (A) Group mean curves for the three injection durations of 2hr, 24 hr or 7 days. The peak is higher for 24 hr injection than for 2 hr, but is lower after 7 days. Sample 1 concentration was lowest at 2 hrs and increased progressively with time. (B) Calculated ratio between sample 3 and sample 1 for individual animals in the study. The fitted regression line suggests that uniform distribution of dextran along ST (3:1 ratio of 1) would typically take about 11 days to achieve. Also shown on the curve is the change of 3:1 ratio with time initially predicted by simulations of the experiment.

Seven-day injection experiments were found to be more challenging. The polyimide cannula was found to have broken where it had been cemented into the bulla in 3 animals and the pump failed in 1 animal. Successful injections and sampling were achieved in 3 of the 7 animals (Fig 5C). In the successfully-injected animals the highest sample of the average was sample 2. Perilymph concentrations after 7 day injections were significantly lower than those with 24 hr injections (2-way ANOVA, Bonferroni t-test, p<0.001) but sample 1 was notably higher than both groups with shorter injection durations. The mean 3:1 ratio was 1.54 (SD 0.54, n = 3). The mean curves for the 3 groups are overlaid in Fig 6A. A progressive increase of apical perilymph concentration (sample 1) as injection time increases is apparent. Fig 6B shows the measured variation of sample 3:1 ratio over time for individual experiments. A regression line fitted to the points indicates that the time for the base-apex longitudinal gradient to subside (to a 3:1 ratio of 1) would require 11 days of injection. For comparison, the predicted decline in ratio, calculated from the curves in Fig 3D is shown as the dotted red line. The experimental data therefore show that achieving uniform distribution of drug along the length of ST with injection through an implanted cannula is even more difficult than we had anticipated.

### Interpretation of 24 hr injection data through simulations

Perilymph measurements following prolonged injections through the implant can be used to improve the accuracy of simulations that were previously based on limited-duration (2–3 hr) studies with injections through glass pipettes that were sealed into the otic capsule. We were initially interested in whether simulations could better fit the new data through parameter adjustments or whether fundamental changes in the underlying processes of the simulations were required.

**Fig 7:**
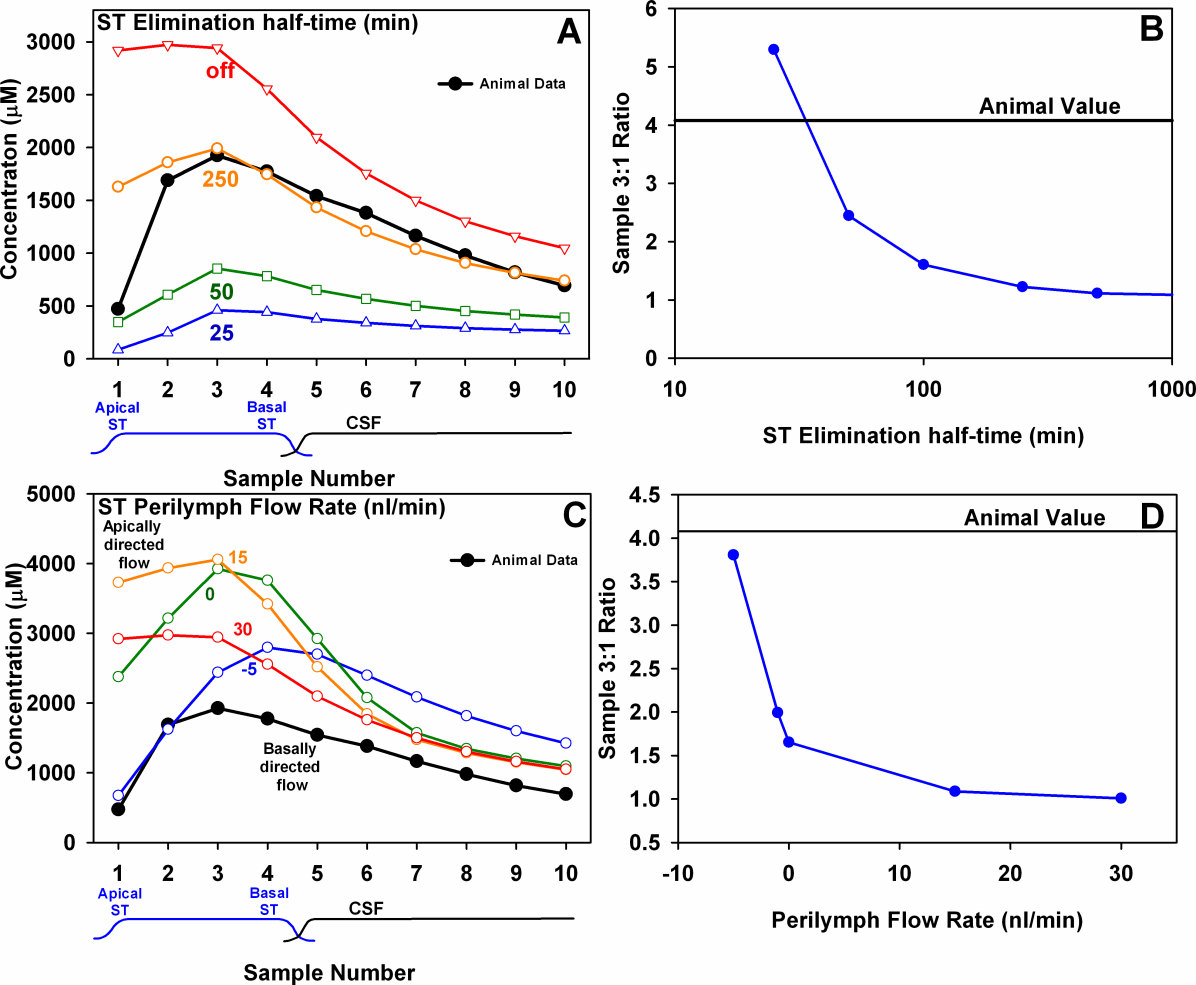
Inability of simulation parameter changes to account for measurements after 24 hrs injection. Upper row (A, B) shows how calculated sample concentrations would be influenced by varying the elimination rate from ST. Concentrations of samples 1 and 2 are decreased as elimination is increased, indicating a greater gradient along ST. However, in order to achieve the measured ratio between samples 3 and 1 (B), elimination must be so fast that the dextran concentrations of all samples are reduced well below what is measured (A). Lower row (C,D) shows how calculated sample concentrations are influenced by varying volume flow along ST. Positive flow rates indicate flow towards the apex along ST and negative rate indicate flow towards the base. As CSF entry and resulting apically directed perilymph flow along ST was reduced, sample 3:1 ratio increased (D). As basally-directed flow increases (increasing negative values), the 3:1 ratio approaches the measured value. However, basally directed flow also increases the dextran concentration in samples 4 and 5 originating from the basal turn (C) as there is less dilution caused by CSF entering ST. Changing flow rates cannot therefore produce the measured 3:1 ratio and the measured curve shape around samples 4 and 5. These calculations show that the measured results cannot be explained by adjustments of elimination rate or flow rate in the ear.

An important parameter affecting drug gradients along the scalae is the process of elimination, which represents the loss of solute from perilymph in an irreversible manner, assumed to be directed to blood. For local drug applications, longitudinal gradients along ST become larger as elimination increases. If elimination occurs faster than the drug diffuses, the drug may never reach the cochlear apex in appreciable concentration and longitudinal gradients will remain high over time. Although previous studies indicated dextran elimination was slow, we considered whether a higher elimination rate could account for the results. The influence of elimination on calculated sample curves is shown in Fig 7. Fig 7A shows the calculated samples for 24 hr applications, with their corresponding sample 3:1 ratios shown in Fig 7B. Increasing elimination from ST increased the longitudinal gradient at 24 hours, as measured by the increase of the sample 3:1 ratio. With elimination half time at about 30 min the 3:1 ratio was comparable to that of the average measured curve. However, elimination at this rate caused all calculated concentrations to be reduced, with sample 3 only at ~29% of that measured. Changing elimination rate cannot therefore simultaneously explain both the magnitude of the longitudinal gradient and the amount of dextran measured in samples after 24 hr injections.

A second factor influencing drug gradients along ST is the ongoing slow flow of CSF entering ST at the cochlear aqueduct. In prior studies, we found this was the primary cause of dextran decline in the basal turn of ST following injections, with an estimated CSF influx of around 30 nL/min accounting for the decline. Volume flow along ST towards the apex helps move dextran apically, thereby reducing the gradient. We therefore considered whether a lower volume flow rate could result in larger gradients for longer injections. The influence of varying flow rate on sample curves and the 3:1 ratio are shown in Fig 7C and 7D respectively. Reducing flow towards the apex does increase the 3:1 ratio, and including a small basally-directed flow in ST brings the calculated ratio close to that measured. However, changing flow in ST also changes the shape of the sample curve, specifically increasing the concentration of samples from basal regions (samples 4 and 5) as the influx of CSF is reduced. Reducing CSF influx and increasing movement from the injection site towards the aqueduct both act to increase dextran concentration in the most basal part of ST. The calculations show that it is not possible to account for the measured drug gradients and concentration of samples originating from the basal turn by varying flow rate. Simple parameter modifications of the simulations apparently cannot account for the measured dextran distributions after 2 hr and 24 hr injections.

**Fig 8:**
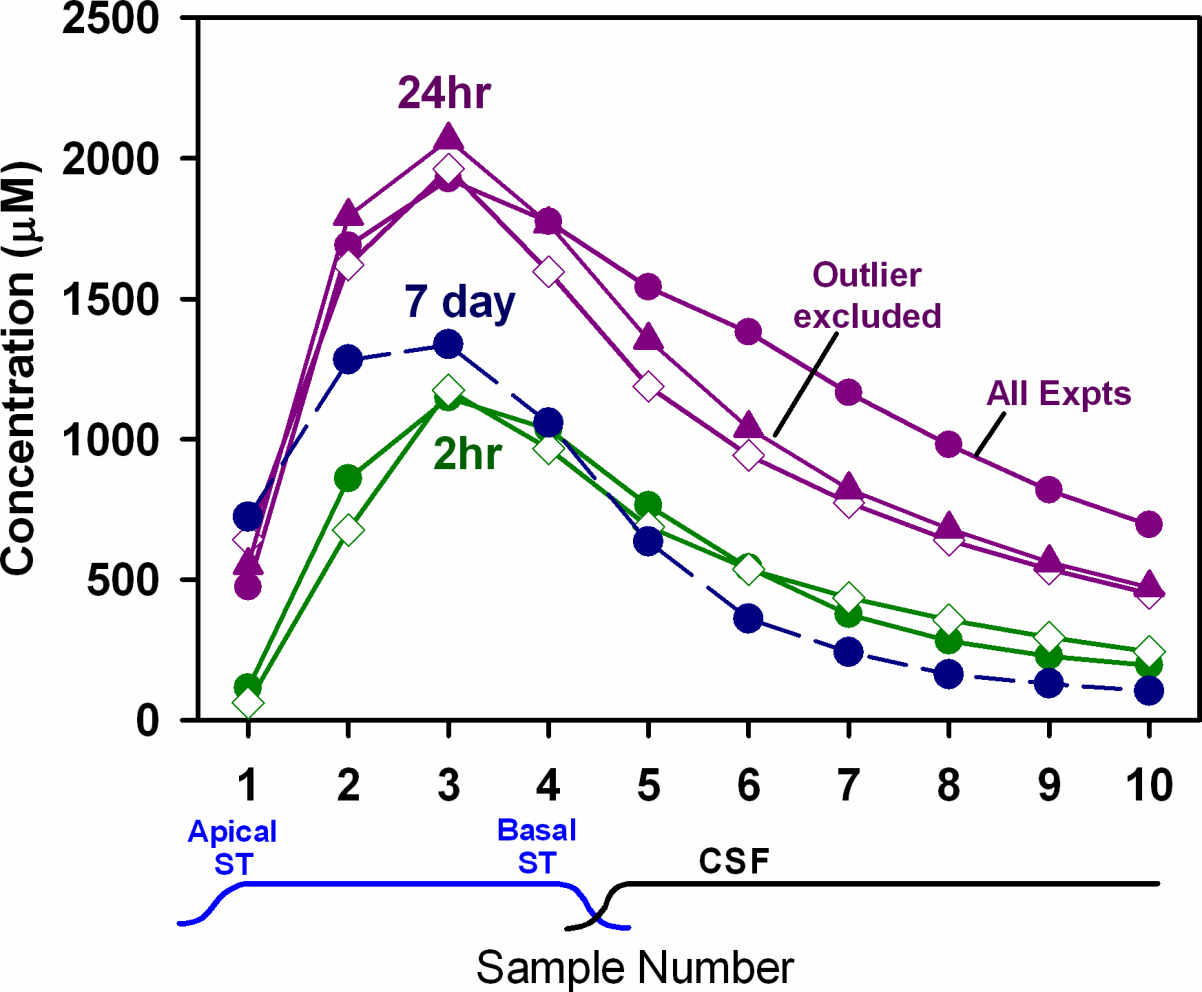
Measurements compared to simulations of the experiments. Open symbols: Calculated sample curves for 2 hr (green) and 24 hr (purple) injections using the same pharmacokinetic parameters. In these simulations we implemented a base-apex gradient of elimination (800 min half time at the base, transitioning to 30 min half time at the apex) and a base-apex gradient of volume flow (0.06 μL entering at the base, transitioning to zero at the apex). This scenario permitted the gradients along ST shown by samples 1–3 to be approximated after 2hr and 24 hr injections. Solid symbols: Measured group average curves. In the case of 24 hr injections the measured curve is also shown excluding one animal that had an abnormal decline in the later samples, which largely accounted for the difference between the measured and calculated curves in samples 6–10. The group mean for 7 day injections is also shown, but the calculated curve for 7 days of injection was identical to that at 24 hours (purple, open symbols) so it has been omitted.

By making substantial changes to some of the processes in the simulations we were able to approximate both the 2hr and 24hr sample data with a single set of parameters, as shown in Fig 8. In these calculations, we added base-apex gradients in both the rate of elimination and in the rate of longitudinal volume flow. Elimination was set to be faster from the apex (half time 30 min; keeping concentrations there low) transitioning to a slower rate at the base (half time 800 min, allowing concentrations there to become higher). Longitudinal flow was set to be 0.06 μL/min at the base (causing dilution of samples originating there), but declining with distance apically along ST until there was zero flow at the apex. This reduced the apical distribution of dextran. The slowing flow rate with distance could be thought to represent a progressive loss of fluid to some other compartment, such as the vasculature, endolymph, or middle ear through canaliculi in the bony otic capsule. With these gradients included in the simulations, curves close to the measured distribution at 2 hrs and 24 hours could be achieved with the same set of parameters (Fig 8). Simulations with these parameters provide a reasonable representation of dextran levels over time when dextran solution is delivered to the basal turn from an implanted cannula.

### Implantation-induced changes in auditory function

**Fig 9:**
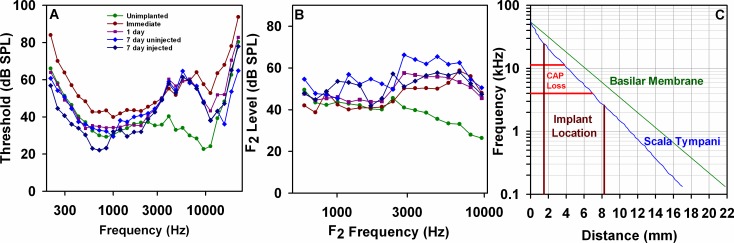
Functional changes caused by implantation. Mean CAP threshold curves (A) and acoustic emission threshold curves (B) for 5 experimental groups with group numbers given below for CAP and emissions respectively. Unimplanted: (Green: 16, 7); Immediately after implantation (Brown: 9, 1); 1 day after implantation with injection (Purple, 5, 5); 7 days after implantation where injection failed (Blue, 4, 4); 7 days after implantation where injection was successful (Dark Blue, 3, 3). The implant location is shown on a frequency/distance plot for the guinea pig (C), adapted to show the relationship with distance along scala tympani (blue curve). The frequency range for significant CAP sensitivity loss corresponds to the middle region of the implant.

Changes in auditory sensitivity as a result of implantation, assessed by CAP thresholds and acoustic emission thresholds are summarized in Fig 9A and 9B respectively. For all implanted groups, loss of CAP sensitivity was most pronounced at around 6.7 kHz where the mean loss was close to 30 dB. CAP thresholds of all implanted groups were significantly elevated compared to unimplanted animals over the range of 4 kHz to 11.3 kHz. (ANOVA, Bonferroni t-tests, p<0.05). Thresholds measured immediately after implantation were significantly elevated across a broader frequency range (ANOVA Bonferroni t-tests, p<0.05, all frequencies except 1.68 kHz to 2.38 kHz). Acoustic emission thresholds were significantly elevated for 1 day and 7 day groups for frequencies of 4 kHz and higher with significance extending down to 2.8 kHz for the 1 day and 7 day non-injected groups (ANOVA Bonferroni t-tests, p<0.05). Elevation of emission thresholds reached 25 dB at the highest frequencies tested.

The frequency range of CAP threshold measurements and the location of the cochlear implant are compared on a frequency–place map for the guinea pig (from [32]) in Fig 9C. Frequency vs. distance along the basilar membrane is shown as the green line, transformed to distances along ST in the blue line. The implant was inserted about 1.5 mm from the base of ST and extended 6.25 mm up the scala, as indicated by the brown lines. Red lines show the range of significant CAP threshold elevation, which corresponds to the middle region of the cochlear implant.

### Morphologic studies

**Fig 10:**
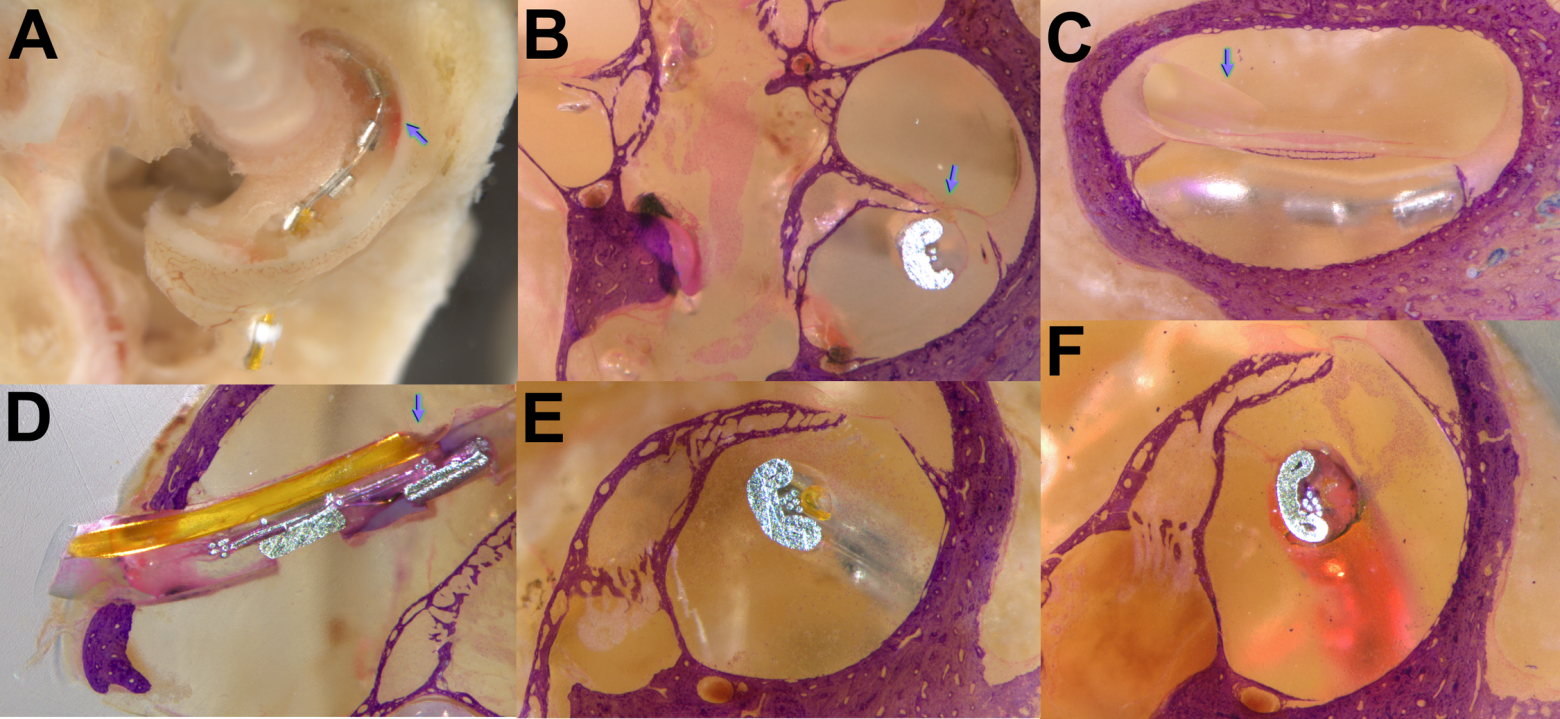
Histological assessments of implanted cochleae. (A) 7 day; dissection opening scala vestibuli and removing the organ of Corti. Reddening on the wall of ST is apparent (arrow). (B) 7 day; implant contacting spiral prominence near basilar membrane (arrow). (C) 7 day; Reissner’s membrane normal (arrow), no indication of endolyphatic hydrops. (D) 24 hr; tight cochleostomy and unobstructed cannula outlet (arrow). (E) 24 hr; electrode at the region of cannula outlet near the middle of ST. Some particulate material is present in the fluid space of ST. (F) 24 hr; another specimen with plume of particulate material in the fluid of ST.

After fluids sampling and electrophysiological measurements, the ears of animals undergoing 1 day or 7day injections with the implant still in place were fixed for histologic study. In Fig 10A the location of the electrode in ST is seen after SV and the organ of Corti had been dissected away. There is some reddening of the lateral portion of ST (arrow) near the location of electrodes 4 and 5 (electrodes 2 to 6 are visible in this image). The location of the cannula outlet is visible just apical to electrode 2. In Fig 10B to 10F the ear was embedded in epoxy resin, the surface ground and stained with eosin and toluidine blue. In Fig 10B the implant is seen to be in contact with the spiral prominence near the medial side of the basilar membrane. Fig 10C shows a 7-day specimen with the implant visible in ST and the endolymphatic space visible. Reissner’s membrane appears normal (arrow) with no indication of endolymphatic hydrops. In Fig 10D the specimen was ground parallel to the implant allowing the cochleostomy, implant and cannula to be visualized. The electrode is seen to be tightly seated in the cochleostomy. The cannula outlet is clear with no occlusion. In Fig 10E a 24 hr injected specimen has been ground orthogonal to the implant. The yellow tip of the cannula is visible, showing no indication of occlusion. At this location, the implant is near the center of ST and does not contact the sensory organ. Particulate material is present in ST around the implant. Fig 10F shows another 24 hr specimen with particulate matter in the perilymphatic space surrounding the implant.

## Discussion

These are the first studies in which the distribution of a substance along ST has been demonstrated during injection through a cannula incorporated into a cochlear implant. The perilymph measurements found that after 2 hr injections, large concentration gradients were present along ST. Contrary to our expectations based on prior pharmacokinetic data, the gradients remained and were still substantial, though reduced, after injections of 1 day duration. They had almost disappeared after injections for 7 days. Based on a fitted line to the sample 3:1 ratio data, it was estimated that an average of 11 days of injection would be required for the gradients to disappear. As the FITC-dextran molecule (4000 FW) is relatively large and in prior studies did not readily pass through cochlear boundaries, longitudinal gradients after prolonged periods of injection would not be expected for this type of molecule. Gradients are typically larger and more persistent for smaller molecules that permeate cochlear boundaries more readily.

Quantitative interpretation of the sustained longitudinal gradients by the simple physical processes that our simulation program is based on proved to be extremely difficult. In order to maintain a low concentration for the apical half of ST (from which sample 1 originates) we considered incorporating a progressively diminishing flow rate along ST (limiting how much dextran was carried apicalwards) and a gradient of elimination, in which elimination occurred rapidly from perilymph in apical regions. Although the simulations with these parameters provided a good representation of the perilymph measurements for injections of up to 24 hours duration, we have serious concerns about the validity of the solution. A high rate of elimination from the apex, needed to account for the results of injection from an implant, is not consistent with the findings from prior studies in which dextran was delivered from glass pipettes sealed into the bony walls of the scalae [30]. In those studies, dextran was lost rapidly from basal regions of ST, due perilymph-CSF interactions, but was retained well in apical regions of the cochlea over a 2-hour period. There are a number of possible explanations for the difference in observations. In prior short-term studies, dextran was injected from a syringe/pipette system with noncompliant rigid walls, while in the current experiments the implant cannula was connected to the pump with mechanically compliant silicone tubing. The increased compliance may allow greater respiratory pressure-induced fluid oscillation across the cochlear aqueduct, perhaps impacting perilymph kinetics. This explanation is thought unlikely as the greatest influence would have been in the basal turn, rather than the apex. Alternatively, over the time period of days in this study, the immune response of the ear to the implant could be influencing perilymph kinetics. All the simulations presented here have assumed parameters remain constant over time. In mice injected with lipopolysaccharide as a model of sepsis, the immune response has been shown to degrade the integrity of the blood-labyrinth barrier [33]. In our analysis, increasing permeability of the barrier over time would increase elimination and potentially explain the persistent gradients for dextran along ST. While this explanation is an attractive hypothesis, more data are required in support of it. Nevertheless, an implant-induced compromise of the blood-labyrinth barrier may explain why extended used of systemic steroids was found to help preserve hearing [22]. This could be accounted for if a compromised blood-labyrinth barrier gives systemic steroids have greater access to perilymph of the implanted ear compared to unimplanted ears. Thus, while the simulation parameters derived from our analysis still provides a quantitative representation of perilymph marker levels with time for up to 24 hours, more complex explanations appear likely. To date, we have not attempted simulations with time varying parameters but realize this may be required if the concept becomes better supported by additional data.

We found that fluid leakage at the cochleostomy site had a large influence on the perilymph levels of dextran achieved. With injections at 100 nL/min, the peak sample concentration moved from sample 4 (without leakage) to sample 3 when leakage was present. This is due to the increased washout from the basal turn caused by CSF entry through the cochlear aqueduct when leakage is present, decreasing the concentration in samples originating from the basal region of ST. Although this presents a significant problem for animal experiments, the influence of fluid leakage at the cochleostomy will be far less in humans. CSF pressure in the human at the level of the cochlear aqueduct is typically negative while sitting or standing. The cochlear aqueduct is longer and narrower in humans compared to rodents [34] and ST volume is larger in the humans, so the influence of volume influx on ST kinetics will be lower. If efficacy and toxicity are established in animals as the dose is varied, both may be underestimated compared to the human as the drug washout present in animals with leaking cochleostomies will not occur to the same degree in humans with leaking cochleostomies.

Placement of an implant in a normally-hearing ear resulted in a hearing loss for frequencies from 4 to 11.3 kHz which averaged as high as 30 dB. Lower and higher frequency responses were less affected or not significantly affected. The hearing loss was stable over the 7-day duration of this study. The absence of a time course of change (such as a progressive deterioration or progressive improvement with time), suggests it does not arise from gross damage to the organ of Corti or spiral ligament, or from mechanical overstimulation during implantation, comparable to noise exposure. Instead, the loss is more compatible with a restriction of basilar membrane motion, perhaps by contact with the implant, or with local disturbance of some other aspect of transduction, such as the electrically insulating silicone disturbing normal current or ion flow in the fluids. The similarity of acoustic emission and CAP changes supports the idea that function of the outer hair cells is being impeded in some manner. Although we did not quantify endolymphatic changes in this study we saw no evidence of endolymphatic hydrops in histologic specimens (Fig 10) and no indication of low frequency hearing loss (Fig 9). This differs from the report of Smeds et al. [13] that suggested endolymphatic hydrops was prevalent in the first weeks following implantation. The magnitude of the losses found here are comparable to the 20–30 dB losses reported previously based on ABR and CAP recordings in implanted guinea pigs with initially normal hearing [1,2,35,36]. However, contrary to the prior studies we found threshold elevations to be limited to the implant location with no significant influence on high and low frequency responses. The functional changes measured here were limited to the initial 7-day period after implantation in normally-hearing animals with no electrical stimulation and therefore have limited relevance to the progressive changes occurring over much longer time courses with ongoing electrical stimulation. Nevertheless, studying the origins, mechanisms and progression of hearing loss in the implanted ear will provide a better understanding of implantation-induced hearing loss.

Histologic analysis using a technique that allowed the electrode to remain in place showed there was no indication of occlusion at the cannula outlet. This is probably because injection occurred continuously throughout the study period. In specimens injected for 24 hrs we observed a plume of particulate material in the fluid space of ST associated with the implant. The resolution of the histologic technique used here did not allow the nature of these particles, cellular or otherwise, to be identified. Others have reported foreign body giant cells, macrophages and lymphocytes associated with the implant when examined years [37], 4 weeks [38] or 3 days [39] after implantation. Our observations suggest a strong morphologic response to implantation can occur within 24 hours of the procedure. It is probable that the response in the perilymphatic space of ST would not be resolved if the implant was removed prior to processing.

All experiments in this study used a large (4000 MW) dextran marker which does not readily pass through cell membranes and is retained in perilymph better than all other substances tested to date [30]. The low rate of elimination acts to minimize longitudinal gradients along the scala. If a substance is eliminated from perilymph more rapidly, gradients are larger and may be maintained longer over time. An example is dexamethasone which is eliminated from ST with a half-time of 22 min [40]. When applied locally to the base of the cochlea, dexamethasone is lost rapidly as it diffuses along the scala to an extent where a steady state is established and where it never reaches the apex in appreciable concentrations. We therefore expect that if gradients for dextran are present along the scala, then even larger gradients would be present for other substances that are eliminated more rapidly than dextran.

## Supporting information

S1 File Numeric data of graphic figures are provided as excel spreadsheets. (ZIP)
